# Selective and sensitive recognition of Zn^2+^ by a dansyl-derived peptide sensor

**DOI:** 10.1039/d6ra00876c

**Published:** 2026-06-01

**Authors:** Alexandre Bianchi, Miriam Gaal, Priscilla S. Brunetto, Claudia Tringali, Katharina M. Fromm

**Affiliations:** a Univ. Fribourg, Department of Chemistry, National Center of Competence in Research Bio-inspired Materials Chemin du Musée 9 1700 Fribourg Switzerland katharina.fromm@unifr.ch

## Abstract

A dansyl-derived peptide sensor, Dansyl-HGHW (D_1_), was designed and investigated for the selective and sensitive recognition of Zn^2+^. The selectivity of D_1_ toward Zn^2+^ among twelve metal ions (Na^+^, K^+^, Ag^+^, Mg^2+^, Ca^2+^, Mn^2+^, Ni^2+^, Cu^2+^, Zn^2+^, Cd^2+^, Al^3+^, and Fe^3+^) was evaluated using fluorescence measurements at an excitation wavelength of 290 nm, showing a pronounced preference for Zn^2+^. The influence of various Zn^2+^ counterion salts (NO_3_^−^, AcO^−^, I^−^, SO_4_^2−^, Cl^−^, ClO_4_^−^) on the sensing performance of D_1_ showed no significant influence. Interference studies indicated that the majority of metal ions did not affect Zn^2+^ detection, except for Ni^2+^ and Cu^2+^, which interfere with the sensing response. pH-dependent fluorescence studies of D_1_ in the presence of Zn^2+^ showed that effective Zn^2+^ coordination occurs exclusively above the imidazole's p*K*_a_, under basic conditions (pH 8–12). Binding studies revealed a strong interaction between D_1_ and Zn^2+^ with a binding constant of 1.46 × 10^5^ M^−1^ and a limit of detection of 47.15 nM. Furthermore, binding interaction analysis using Job's plot indicated the presence of successive 1 : 1 and 3 : 2 metal-to-ligand stoichiometry. Cytotoxicity studies revealed that D_1_ is non-toxic to L-929 fibroblast cells over the tested concentration range (12.5–200 µM). Additionally, cell imaging studies have demonstrated the efficacy of D_1_ in detecting intracellular Zn^2+^. These results indicate that D_1_ is a promising peptide-based fluorescent sensor for selective Zn^2+^ detection.

## Abstract 2

A dansyl-derived peptide sensor, Dansyl-HGHW (D_1_), was designed and investigated for the selective and sensitive recognition of Zn^2+^.

## Graphical abstract

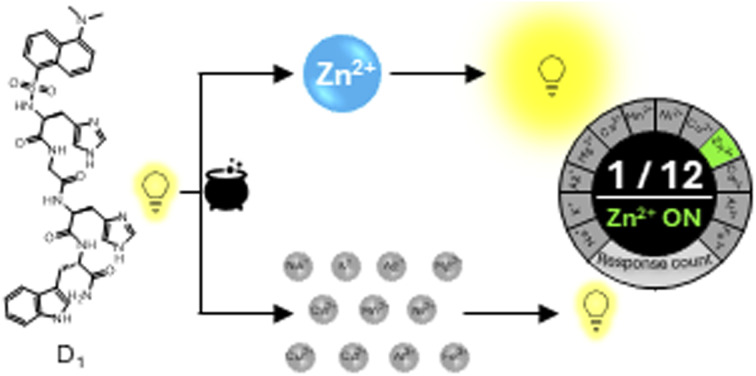

## Introduction

Zinc (Zn^2+^) is the second most abundant transition metal in biological systems and plays a crucial role in cellular processes.^1,2^ Zn^2+^ is present in the active sites of key enzymes, including hydrolases, carbonic anhydrase, alcohol dehydrogenase, and various synthases, where it stabilizes reaction intermediates and accelerates fundamental biochemical transformations.^1–4^ Beyond its catalytic role, Zn^2+^ contributes to the stabilization of protein architectures, notably in zinc-finger domains that regulate gene transcription, RNA/DNA recognition, and protein–protein interactions.^1,2,5^ In addition, Zn^2+^ is required for DNA repair enzymes and proteins involved in genome maintenance.^2^ The combination of catalytic and structural functions makes Zn^2+^ essential for cellular homeostasis, and disturbance in its intracellular concentration can lead to pronounced biological dysfunctions.^2,3^

These considerations underline Zn^2+^ as a pivotal bioinorganic target and justify the growing interest in the development of sensitive, selective, and biocompatible molecular tools for its detection in biological environments. Among various approaches, peptide-based sensors have emerged as particularly promising candidates due to their excellent biocompatibility and the ability to engineer amino acid sequences for efficient metal-ion binding. Residues such as histidine (His, H), cysteine (Cys, C), aspartic acid (Asp, D), and glutamic acid (Glu, E) are often used for this purpose.^2,5–7^

This design strategy is supported by previous studies on dansyl-based peptide sensors (Table 1).^8–13^ Short peptides functionalized with an N-terminal dansyl fluorophore and a C-terminal amide group have been shown to be effective for the fluorescence detection of Zn^2+^ and other biologically relevant metal ions.^8,9^ For instance, Zn^2+^ selective probes such as Dansyl-HPGHWG-NH_2_ and Dansyl-CPGH-NH_2_ exhibited limits of detection (LOD) of 97 nM and 82 nM in HEPES buffer, respectively.^8,9^ It is interesting to note that these sequences contain histidine residues and, in one case, a tryptophan unit, thereby highlighting the importance of coordinating amino acids and aromatic residues for achieving efficient metal binding and fluorescence enhancement. Furthermore, related systems have been reported for Cd^2+^, Hg^2+^, and Cu^2+^ detection, highlighting the versatility and adaptability of this molecular system.^10–12^

**Table 1:** Overview of other published dansyl-based peptide sensors that have been used to detect several metal ions

Sequence	Detection media	Selectivity	LOD [nM]	References
Dansyl-HPGHWG-NH_2_	HEPES buffer	Zn^2+^	97	Wang *et al.* (2015)^8^
Dansyl-CPGH-NH_2_	HEPES buffer	Zn^2+^	82	Wan *et al.* (2018)^9^
Dansyl-PGC-NH_2_	HEPES buffer	Cd^2+^	12.4	Deng *et al.* (2025)^10^
Dansyl-ECEW-NH_2_	HEPES buffer	Hg^2+^	23.0	Pang *et al.* (2020)^11^
Dansyl-DG-NH_2_	HEPES buffer	Cu^2+^	1520	Song *et al.* (2023)^12^
Dansyl-HTEHW-NH_2_	Water	Cu^2+^, Hg^2+^, Zn^2+^	37.6, 37.8, 59.4	Zhang *et al.* (2022)^13^

In this study, the HGHW tetrapeptide motif was selected as an effective Zn^2+^-binding sequence. The two histidine residues serve as the primary coordination sites, glycine (Gly, G) provides conformational flexibility and minimizes steric constraints, and the terminal tryptophan (Trp, W) acts as a Förster Resonance Energy Transfer (FRET) donor to the dansyl fluorophore.^14^ Upon Zn^2+^ coordination, this energy-transfer pathway leads to a significant enhancement of fluorescence emission, thereby providing an efficient signal amplification mechanism.^8,15,16^

**Fig. 1:**
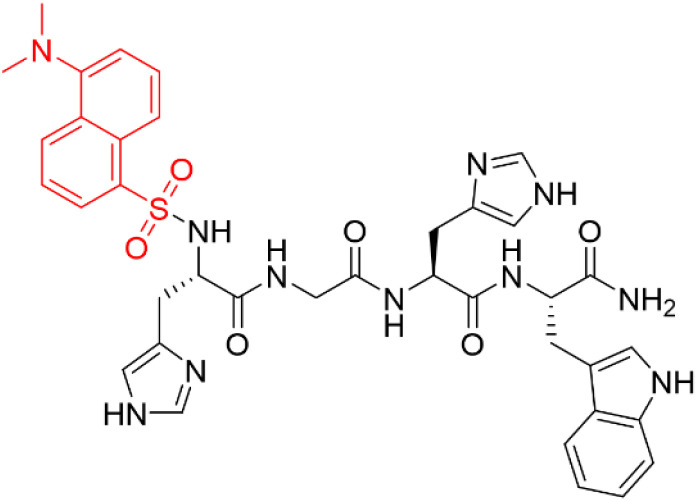
Chemical structure of D_1_. The dansyl group is highlighted in red, while the HGHW motif is shown in black.

Consequently, this study presents the design and investigation of Dansyl-HGHW-NH_2_ (D_1_) (Fig. 1), as a promising fluorescent sensor for selective and sensitive Zn^2+^ detection in both aqueous and biological media.

## Experimental part

### Materials, chemicals, and instruments

The following chemicals were procured from various commercial suppliers: Fmoc (9-fluoromethoxy-carbonyl) protected H-Rink Amide ChemMatrix resin (loading 0.42–0.47 mmol g^−1^), dichloromethane (DCM), piperidine, *N*,*N*-dimethylformamide (DMF), Fmoc (9-fluoromethoxy-carbonyl) protected amino acids (Fmoc-His(Trt)-OH, Fmoc-Gly-OH, Fmoc-Trp(Boc)-OH), O-(1*H*-6-Chlorobenzotriazol-1-yl)-1,1,3,3-hexafluorophosphate (HCTU), 1*H*-1,2,3-Benzotriazol-1-ol hydrate (HOBt), *N*-Ethyl-*N*-(propan-2-yl)propan-2-amine (DiEA), 1-methylpyrrolidin-2-one (NMP), Acetic anhydride (Ac_2_O), 9-anthracene carboxylic acid, dansyl chloride, Trifluoroacetic acid (TFA), Tri(propan-2-yl)silane (TIPS), Ethane-1,2-dithiol (EDT), Diethyl ether, Acetonitrile (ACN), 4-(2-hydroxyethyl)-1-piperazineethanesulfonic acid (HEPES), and Dimethyl sulfoxide (DMSO). All solvents used were analytical grade.

Stock solutions of nitrate salts of the following ions were prepared in bidistillated water: Na^+^, K^+^, Ag^+^, Mg^2+^, Ca^2+^, Mn^2+^, Ni^2+^, Cu^2+^, Zn^2+^, Cd^2+^, Al^3+^, and Fe^3+^. In addition, stock solutions of various zinc salts, including acetate, iodide, sulfate, chloride, and perchlorate, were also prepared.

The peptide was purified by semi-preparative reverse-phase HPLC using a NucleoDur™ C18 HTec column on a Waters Delta 600 system. ESI-MS analysis was performed on a Bruker Esquire HCT spectrometer. The freeze-dried sample was obtained using a Christ Alpha 1–2 LDplus lyophilizer. The concentration of the peptide stock solution was determined using a PerkinElmer Lambda 25 UV-vis spectrophotometer according to the Beer–Lambert law. All fluorescence spectra were recorded on a PerkinElmer LS50B fluorescence spectrometer. The lifetime was measured using an Edinburgh Photonics EPL-405. All pH values were measured using a Mettler Toledo InLab®NMR pH meter. Cytotoxicity assays were measured using a TECAN Infinite® 200 Pro microplate reader.

### Solid-phase synthesis of D_1_

D_1_ was synthesized manually using standard Fmoc solid-phase peptide synthesis (SPPS) under continuous agitation at room temperature.^17,18^ The dry Fmoc-protected H-Rink Amide ChemMatrix resin was first swollen in DCM for 60 minutes. Fmoc deprotection was carried out twice using 20% piperidine in DMF for 10 minutes each. The coupling of Fmoc-protected amino acids (Trp, His, and Gly) was performed in DMF using HCTU and as coupling agents and in NMP as an organic base for 60 minutes. Unreacted amines were capped with a mixture of Ac_2_O in DMF and DiEA in NMP for 20 minutes to prevent undesirable side reactions.

After the peptide sequence was assembled, the chromophore (dansyl chloride) was attached to the N-terminus under standard coupling conditions. The resin was then washed with DCM before cleavage. Peptide cleavage and sidechain deprotection were achieved using a mixture of 95.5% TFA, 1.5% bidistillated water, 1.5% TIPS, and 1.5% EDT for 2 hours at room temperature. The cleaved peptides were filtered, and the filtrate was precipitated with cold diethyl ether. The crude peptides were collected by centrifugation at 7500*g* for 6 minutes, dried, and purified by semi-preparative reverse-phase HPLC using a linear gradient from 95% to 70% of solvent A in B over 25 minutes at 5 mL min^−1^ (solvent A: 0.1% TFA in Milli-Q water; solvent B: 0.1% TFA in ACN) (Fig. S1). The purified and characterized peptide (Fig. S2) was then lyophilized for 2 days.

### Fluorescence measurements

A stock solution of D_1_ was prepared in bidistillated water and stored at 4 °C. All fluorescence measurements were performed in a 1 cm quartz cuvette using a D_1_ concentration of 1 × 10^−5^ M in 20 mM HEPES buffer (pH 7.4–7.5) at 25 °C. The final volume was adjusted to 2.5 mL with bidistillated water. The fluorescence emission spectra were recorded in the presence of various nitrate salts solutions of Na^+^, K^+^, Ag^+^, Mg^2+^, Ca^2+^, Mn^2+^, Ni^2+^, Cu^2+^, Zn^2+^, Cd^2+^, Al^3+^, and Fe^3+^, as well as in the presence of different zinc salts solutions containing NO_3_^−^, AcO^−^, I^−^, SO_4_^−^, Cl^−^, and ClO_4_^−^ counterions.

The excitation of D_1_ at 280 nm was not feasible because the second-order harmonic of the excitation source produced a signal that interfered with the dansyl emission band (*λ*_em_: 540 nm). To eliminate this spectral overlap, the excitation wavelength for D_1_ was shifted to 290 nm, which primarily excites the tryptophan residue. Additional experiments were performed by directly exciting the dansyl group at 340 nm (more details in the SI, Fig. S4–S8).

### Determination of binding constants

The association constant was determined from the fluorescence titration curve using a non-linear fitting model described by the following equation: *y* = (1 + e^−*s*(*X*−*X*_0_)^)^−1^, where *y* represents the normalized fluorescence response *I* − *I*_0_/*I*_max_ − *I*_0_, *x* is the concentration of the metal ion, *x*_0_ denotes the midpoint concentration corresponding to half-maximal response, and *s* is the slope parameter.^19,20^ The association constant (*K*_a_) was then calculated as: *K*_a_ = (*X*_0_)^−1^.

### Limit of detection

The limit of detection (LOD) for the peptide-metal ion system was determined by measuring 10 times the fluorescence signal of D_1_ in the absence of metal ions to calculate the standard deviation (*σ*). Subsequently, a fluorescence titration was performed by adding increasing concentrations of Zn^2+^, resulting in a linear relationship between fluorescence intensity and Zn^2+^ concentration. The LOD was calculated using the following formula: LOD = 3.3*σ*/*k*, where *k* is defined as the slope of the plot of emission intensity *versus* metal ion concentration.

### Cytotoxicity assays

L-929 fibroblasts cells (NCTC clone 929, ATCC® CCL-1™, American Type Culture Collection, Manassas, VA, USA) were cultivated in RPMI-1640 medium, supplemented with GlutaMAX, 10% heat-inactivated foetal bovine serum, 1% sodium pyruvate, 1 mM non-essential amino acids solution, 1% antibiotics (penicillin–streptomycin), at 37 °C in a 5% CO_2_ humidified atmosphere. Cells were seeded at a density of 2 × 10^3^ cells per well into 96-well plates and incubated for 24 h to allow adhesion. Then, cells were treated with various concentrations of the test compound (12.5, 25, 50, 100, and 200 µM) for 24 h. Cell viability was assessed using the MTT assay. The resulting formazan crystals were solubilized with DMSO, and absorbances were measured at 570 nm. Experiments were performed in triplicate with two independent replicates.

### Cell imaging

L-929 fibroblast cells (NCTC clone 929, ATCC® CCL-1™, American Type Culture Collection, Manassas, VA, USA) were cultivated as described above. Cells were seeded at a density of 6 × 10^3^ cells per well into a µ-slide 8-well chamber and incubated for 24 h to allow adhesion. Then, cells were treated with the test compounds (10 µM) for 30 min at 37 °C in a humidified atmosphere containing 5% CO_2_. Cell imaging was performed using a Leica STELLARIS 8 FALCON (*λ*_ex_: 405 nm, *λ*_em_: 500–650 nm).

## Results and discussion

### Study of selectivity of D_1_

**Fig. 2:**
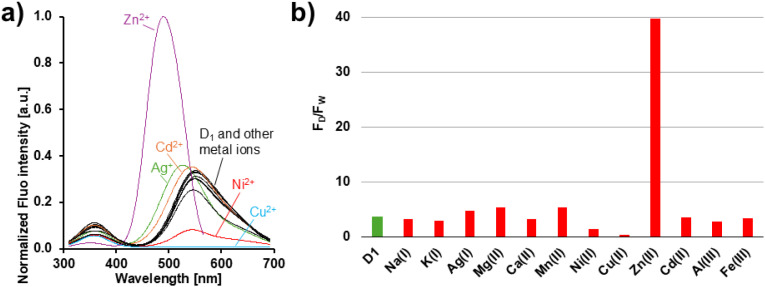
(a) Voigt-deconvoluted and normalized fluorescence spectra of D_1_ (1 × 10^−5^ M) in HEPES buffer (20 mM, pH 7.4–7.5) at 25 °C, *λ*_ex_ = 290 nm. (b) Corresponding histogram. D_1_ (green bar), D_1_ + 3 eq. of the indicated metal ions (red bars). *F*_D_ and *F*_W_ represent, respectively, the dansyl and the tryptophan fluorescence.

As illustrated in Fig. 2, the selectivity of D_1_ toward various metal ions was evaluated by recording fluorescence emission spectra (*λ*_ex_: 290 nm) in the presence of different nitrate salt solutions (Na^+^, K^+^, Ag^+^, Mg^2+^, Ca^2+^, Mn^2+^, Ni^2+^, Cu^2+^, Zn^2+^, Cd^2+^, Al^3+^, and Fe^3+^) in HEPES buffer (20 mM, pH 7.4–7.5) at 25 °C.

Among the ions studied, Cu^2+^ induced a strong fluorescence quenching, consistent with its high affinity for histidine residues and its well-documented ability to efficiently suppress emission.^21,22^ In contrast, Zn^2+^ induced a significant fluorescence enhancement accompanied by a neat blue shift of the emission band (540 nm → 490 nm), indicating the formation of a more emissive D_1_–Zn^2+^ complex. Simultaneously, the tryptophan emission decreased while the dansyl fluorescence increased, consistent with an intramolecular tryptophan-to-dansyl energy transfer (FRET). The other metal ions produced minimal or negligible fluorescence changes under the same experimental conditions. Based on these observations, subsequent investigations were focused on Zn^2+^.

### Study of various Zn^2+^ counterion salts on D_1_

**Fig. 3:**
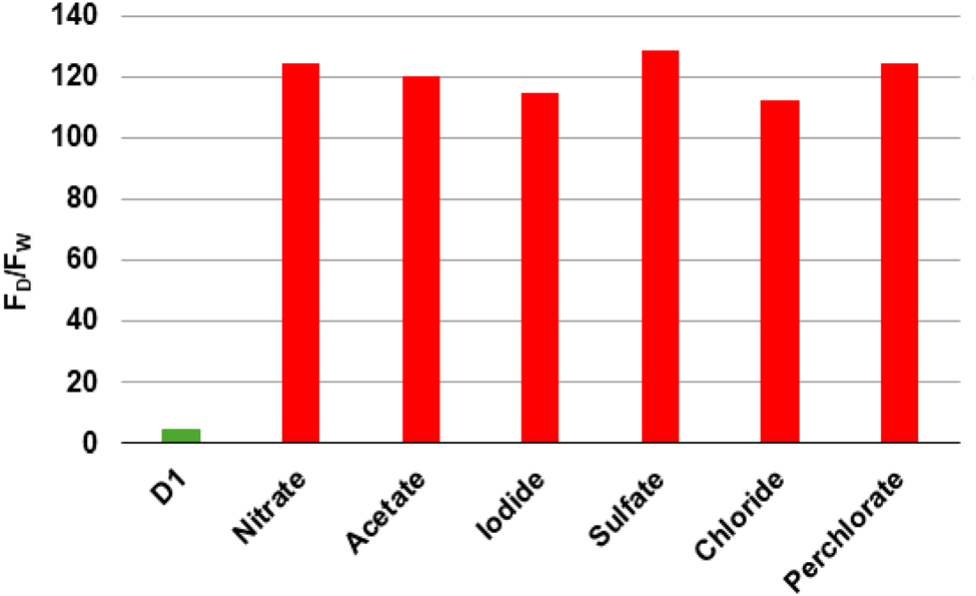
Study of various Zn^2+^ counterion salts on D_1_ (1 × 10^−5^ M) in HEPES buffer (20 mM, pH 7.4–7.5) at 25 °C, *λ*_ex_ = 290 nm. D_1_ alone (green bar), and D_1_ + 3 eq. of the indicated Zn^2+^ salts (red bars). *F*_D_ and *F*_W_ represent, respectively, the dansyl and the tryptophan fluorescence.

As illustrated in Fig. 3, the effect of different Zn^2+^ salts counter ions, including nitrate, acetate, iodide, sulfate, chloride, and perchlorate, on the emission of D_1_–Zn^2+^ complex was investigated. The results indicated that the nature of the counterion has a negligible effect on the fluorescence of the complex. Consequently, Zn(NO_3_)_2_ was selected for all subsequent studies.

### Study of interference by various metal ions

**Fig. 4:**
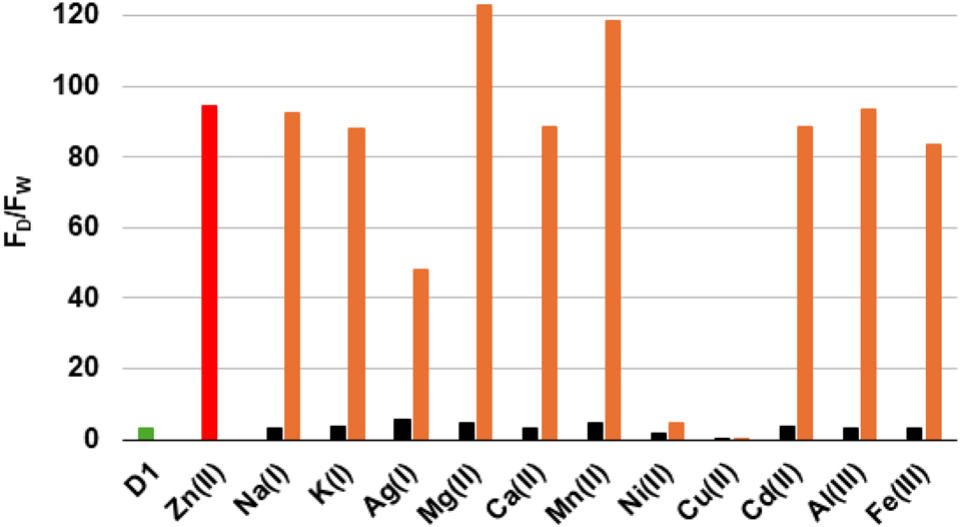
Interference study of various metal ions on D_1_ (1 × 10^−5^ M) in HEPES buffer (20 mM, pH 7.4–7.5) at 25 °C, *λ*_ex_ = 290 nm. D_1_ alone (green bar), D_1_ + 3 eq. of Zn^2+^ (red bar), D_1_ + 3 eq. of the indicated metal ions (black bars), then addition of 3 eq. of Zn^2+^ (orange bars). *F*_D_ and *F*_W_ represent, respectively, the dansyl and the tryptophan fluorescence.

As illustrated in Fig. 4, an interference study was performed to evaluate whether other metal ions can affect the Zn^2+^-induced fluorescence response of D_1_. Initially, D_1_ was incubated with three equivalents of various metal ions (excluding Zn^2+^), and the fluorescence emission was recorded. Subsequently, three equivalents of Zn^2+^ were added to each solution to assess whether pre-bound ions interfered with the Zn^2+^ response.

The results showed that most of the tested metal ions exert a negligible influence on the Zn^2+^-induced fluorescence enhancement of D_1_. Indeed, for Na^+^, K^+^, Ag^+^, Mg^2+^, Ca^2+^, Mn^2+^, Cd^2+^, Al^3+^, and Fe^3+^, the addition of Zn^2+^ restored fluorescence intensities to levels comparable to those observed with Zn^2+^ alone, indicating that these ions do not significantly interfere with Zn^2+^ binding.

Conversely, Ni^2+^ and Cu^2+^ were found to cause significant interference. Indeed, Ni^2+^ induced a significant decrease in fluorescence. Although the addition of Zn^2+^ partially restored the emission, the intensity remained considerably lower than that observed with Zn^2+^ alone. In the case of Cu^2+^, the fluorescence was fully quenched, both before and after the addition of Zn^2+^, indicating that Cu^2+^ has a higher binding affinity for D_1_ than Zn^2+^.

### pH study of D_1_ and Zn^2+^

**Fig. 5:**
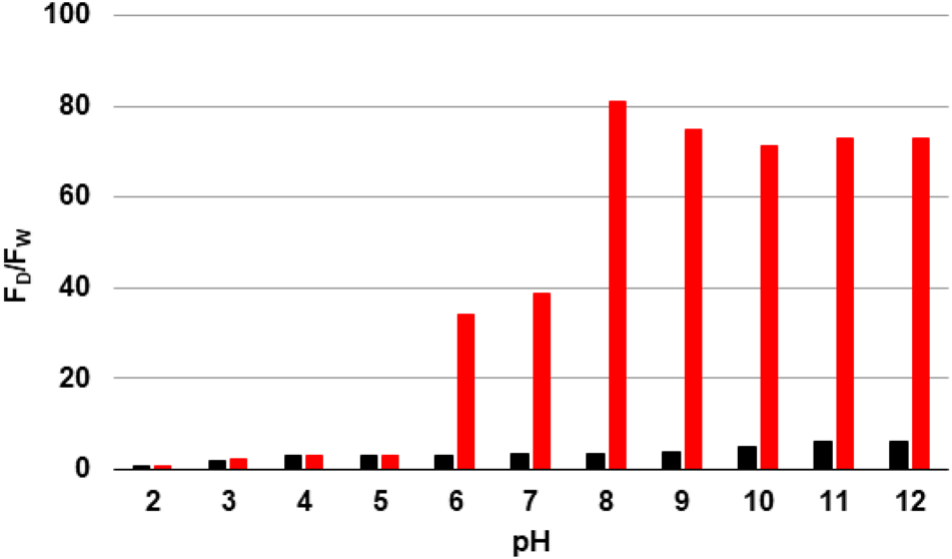
pH-dependent fluorescence response of D_1_ (1 × 10^−5^ M) at 25 °C, *λ*_ex_ = 290 nm. Black bars represent D_1_ alone. Red bars represent D_1_ + 3 eq. of Zn^2+^. The pH was adjusted using HNO_3_ and NaOH solutions, both at 0.1 M. *F*_D_ and *F*_W_ represent, respectively, the dansyl and the tryptophan fluorescence.

As illustrated in Fig. 5, the influence of pH on the fluorescence properties of D_1_ and the D_1_–Zn^2+^ complex was investigated over the pH range 2 to 12. In the absence of Zn^2+^, D_1_ exhibited negligible fluorescence throughout the entire pH range 2–12, indicating pH alone does not significantly affect the emission.

Conversely, in the presence of Zn^2+^, the fluorescence emission exhibited a pronounced dependence on pH. In the presence of an acidic environment (pH 2–5), no substantial emission was detected, which can be attributed to the complete protonation of the histidine side chains (p*K*_a_ ≈ 6.0), preventing Zn^2+^ coordination due to the unavailability of the imidazole nitrogen atoms for metal binding. A slight increase in fluorescence was observed around pH 6–7, corresponding to the partial deprotonation of histidine. At and above pH 8, the histidine side chains are mostly deprotonated, resulting in a strong fluorescence signal upon Zn^2+^ coordination. This coordination induces conformational and structural changes that modulate the spatial distance and orientation between the donor and acceptor units, which enhances the FRET efficiency, resulting in a strong fluorescence emission. These results indicate that effective Zn^2+^ coordination occurs exclusively above the histidine p*K*_a_, under basic conditions (pH 8–12).

### Study of binding interactions between D_1_ and Zn^2+^

**Fig. 6:**
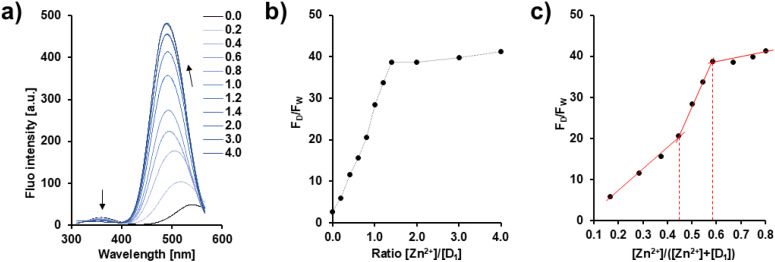
(a) Voigt-deconvoluted fluorescence titration spectra of D_1_ (1 × 10^−5^ M) in HEPES buffer (20 mM, pH 7.4–7.5) at 25 °C, *λ*_ex_ = 290 nm, recorded with increasing amount of Zn^2+^ (from 0 to 4.0 eq.). (b) *F*_D_/*F*_W_ ratio plotted as a function of Zn^2+^ equivalents. (c) Job's plot used to determine the stoichiometry of the D_1_–Zn^2+^ complex. *F*_D_ and *F*_W_ represent, respectively, the dansyl and the tryptophan fluorescence.

The binding interaction between D_1_ and Zn^2+^ was further analyzed, by titrating D_1_ with Zn^2+^ at concentrations of 0, 0.2, 0.4, 0.6, 0.8, 1.0, 1.2, 1.4, 2.0, 3.0, and 4.0 equivalents, in HEPES buffer (Fig. 6a). Upon Zn^2+^ addition, a gradual decrease in the tryptophan fluorescence (*λ*_em_: 360 nm) was observed, accompanied by an increase in the dansyl emission, along with a clear blue shift of the dansyl band (from *λ*_em_: 540 nm at 0 equivalent to *λ*_em_: 490 nm at 4.0 equivalents) (Fig. 6a). These observations indicate a pronounced interaction between the two histidine residues present in D_1_ and Zn^2+^ consistent with a conformational rearrangement that rigidifies the system. This structural change brings the two chromophores into closer proximity, thereby facilitating FRET from tryptophan to the dansyl unit, thereby enhancing dansyl emission.

Analysis of the Job's plot (Fig. 6c) suggests the existence of multiple D_1_–Zn^2+^ stoichiometries, with slope changes that are consistent with the successive formation of 1 : 1 and 2 : 3 D_1_–Zn^2+^ complexes in aqueous solution. The association constant (*K*_a_) for the 1 : 1 complex was estimated to be 1.46 × 10^5^ M^−1^ (*R*^2^ = 0.9927) (Fig. S9). Fluorescence lifetime measurements also support the formation of a D_1_–Zn^2+^ complex. Indeed, D_1_ alone exhibits a lifetime of 4.02 ns, whereas the D_1_–Zn^2+^ complex shows an increased lifetime of 19.18 ns, indicating the stabilization of the excited state upon coordination (Fig. S10). The limit of detection (LOD) for Zn^2+^ using D_1_ was determined to be 47.15 nM (*R*^2^ = 0.992), demonstrating a high sensitivity of the ligand (Fig. S11a).

**Scheme 1:**
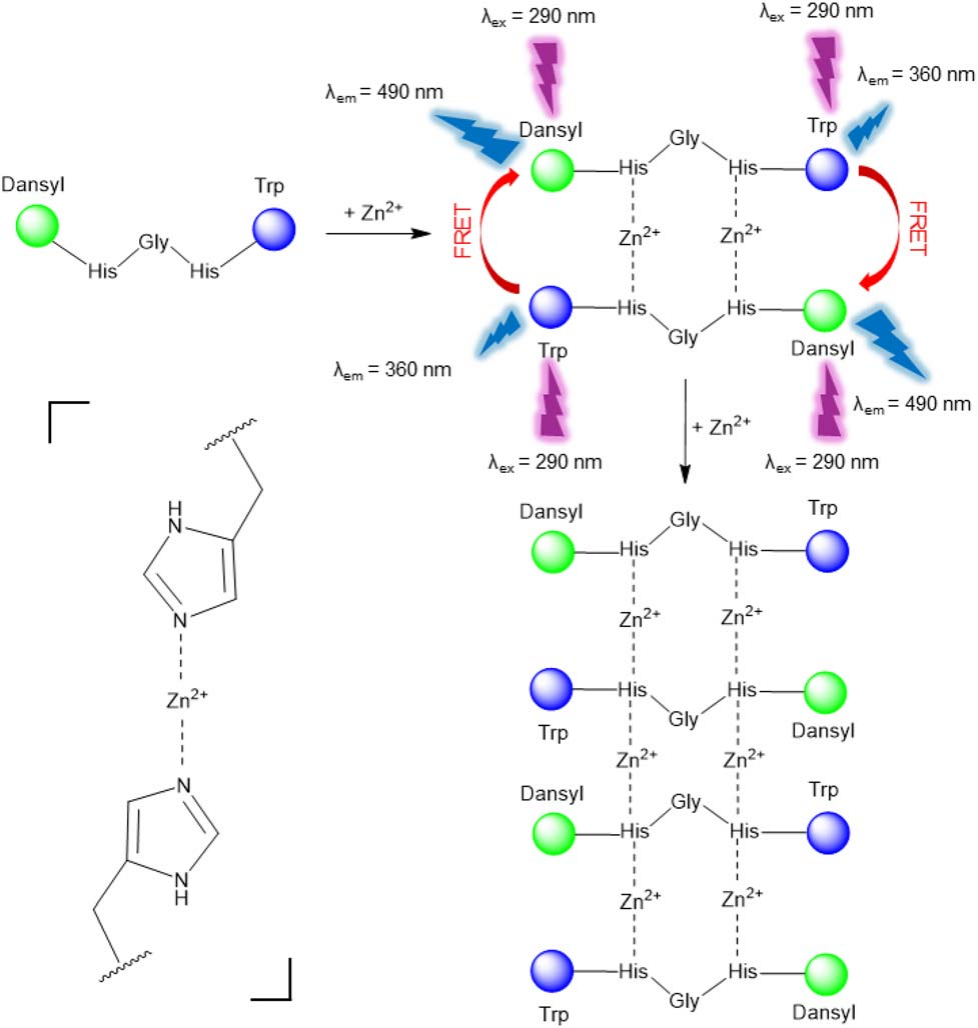
Proposed fluorescence sensing mode of D_1_ for selective Zn^2+^ detection *via* 1 : 1 and 2 : 3 complex formation. Coordination occurs between Zn^2+^ and the imidazole groups of two histidine residues, as schematically represented on the left side of the scheme. Nitrate anions and water molecules were omitted for clarity.

As demonstrated by the ESI-MS data, particularly the peak observed at *m*/*z* = 416.6 (Fig. S3), a 1 : 1 D_1_–Zn^2+^ complex is identified. However, it is hypothesized that this apparent 1 : 1 stoichiometry corresponds to a 2 : 2 complex. In this model, it is proposed that coordination involves two Zn^2+^, leading to a head-to-tail arrangement, bringing the chromophores from different D_1_ molecules into proximity, and thereby favouring intermolecular FRET (Scheme 1). This spatial rearrangement enhances energy transfer between the donor and acceptor, thereby resulting in the observed fluorescence change. Based on our group's previous works showing that Ag^+^ exhibited a preference for binding to the N_ε_ atom of the imidazole ring, it is postulated that Zn^2+^ will also demonstrate a similar binding behavior and be coordinated by the same site in D_1_.^18,23^ In a similar way, the 2 : 3 complex is hypothesized to correspond to a 4 : 6 stoichiometry.

### Cytotoxicity study

**Fig. 7:**
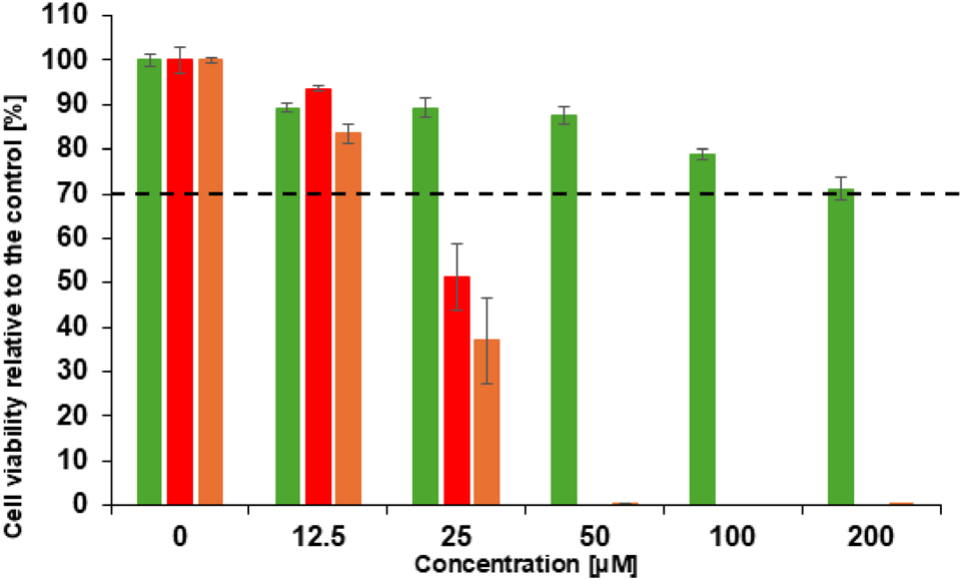
Viability assays on L-929 fibroblast cells in the presence of various concentrations (12.5, 25, 50, 100, and 200 µM) of D_1_ (green bars), D_1_ + 3 eq. of Zn^2+^ (red bars), and Zn(NO_3_)_2_ (orange bars).

As illustrated in Fig. 7, the cytotoxicity studies were performed on L-929 fibroblast cells using the MTT assay. Cell viability was measured after exposure to D_1_ (green bars), D_1_–Zn^2+^ complex (red bars), and Zn(NO_3_)_2_ (orange bars) at various concentrations (12.5, 25, 50, 100, and 200 µM). The results revealed that D_1_ is non-toxic across the tested concentration range, thus indicating its biocompatibility. In contrast, Zn(NO_3_)_2_ alone exhibits significant toxicity, with a marked decrease in cell viability, which was evident at low concentrations tested (12.5 µM). This result is consistent with the literature, which reports that an excess of free Zn^2+^ is cytotoxic. Indeed, an elevated intracellular Zn^2+^ concentration has been demonstrated to induce cellular dysfunction by inducing the production of reactive oxygen species (ROS), oxidative stress, and ultimately leading to the initiation of cell apoptosis.^4,24^

It is important to note that the complexation of Zn^2+^ with D_1_ results in a slight reduction in toxicity, corresponding to an average decrease of approximately 12.5% compared to the free Zn^2+^ salt. Despite the modest nature of this reduction, it suggests that the coordination of Zn^2+^ by D_1_ can partially attenuate the intrinsic toxicity of Zn(NO_3_)_2_. In consequence, these results confirm the biocompatibility of D_1_ and are particularly encouraging for potential biological and biomedical applications of D_1_.

### Cell imaging

**Fig. 8:**
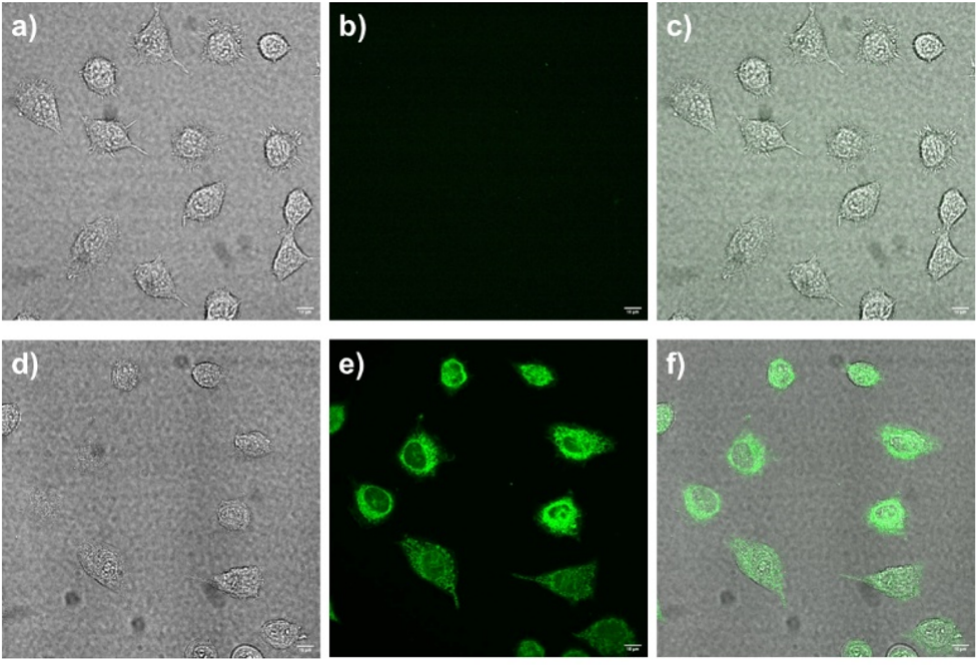
Confocal fluorescence images of L-929 fibroblast cells: bright-field transmission images of L-929 fibroblast cells after incubation with (a) D_1_ alone (10 µM), (d) D_1_ + 3 eq. of Zn^2+^ (10 µM), for 30 min at 37 °C. Fluorescence transmission images (b and e), and merged transmission images (c and f).

As demonstrated in Fig. 8, the cellular absorption and imaging properties of D_1_ in L-929 fibroblast cells were analysed using fluorescence microscopy. Cells treated with D_1_ alone (10 µM) exhibit negligible intracellular fluorescence (Fig. 8b), consistent with the weak intrinsic emission of the probe (Fig. 2).

Conversely, a pronounced fluorescence signal is evident in cells treated with D_1_–Zn^2+^ complex (10 µM). The green colour of cells in Fig. 8e indicates that the D_1_–Zn^2+^ complex can enter into the cellular cytoplasm and confirms that the D_1_–Zn^2+^ complex is suitable for cell staining.

## Conclusions

In this study, we developed D_1_, a dansyl-derived peptide sensor (Dansyl-HGHW), as a selective and sensitive sensor for Zn^2+^ detection. D_1_ exhibits a high degree of selectivity for Zn^2+^ over other metal ions, with negligible influence from different Zn^2+^ counterions. Fluorescence measurements revealed a pronounced blue shift from 540 nm to 490 nm upon Zn^2+^ addition, indicating significant electronic and structural changes in the ligand. pH-dependent fluorescence studies demonstrated that effective Zn^2+^ coordination occurs under basic conditions (pH 8–12), highlighting the crucial role of histidine deprotonation. Binding studies confirmed a strong interaction between D_1_ and Zn^2+^ (*K*_a_ = 1.46 × 10^5^ M^−1^), with analysis suggesting consecutive formation of 1 : 1 and 2 : 3 D_1_–Zn^2+^ complexes, and a low limit of detection of 47.15 nM. Finally, cytotoxicity studies showed that D_1_ is non-toxic to L-929 fibroblast cells, and the bioimaging results are promising.

Collectively, these results establish D_1_ as a robust and promising sensor for Zn^2+^ detection, with potential implications for analytical, environmental, and biological applications. Indeed, the strong selectivity, pronounced fluorescence response, high sensitivity, and good biocompatibility of D_1_ highlight its potential as a promising candidate for the development of peptide-based fluorescent probes for selective Zn^2+^ detection.

## Author contributions

K. M. F. conceptualized the initial idea, obtained competitive funding, and supervised the project in its entirety. A. B. was responsible for the synthesis and fluorescence analysis. M. G. contributes to the project as part of her Bachelor's thesis work. Biological assays were conducted by P. S. B. and C. T. K. M. F., A. B., and P. S. B. participated in the writing and revision of the final manuscript. All authors approved the content and submission.

## Conflicts of interest

There are no conflicts to declare.

## Supplementary Material

RA-016-D6RA00876C-s001

## Data Availability

The data supporting this article have been included as part of the supplementary information (SI). Supplementary information: experimental data. See DOI: https://doi.org/10.1039/d6ra00876c.
