# Supplementary material for: Selective and sensitive recognition of Zn2+ by a dansyl-derived peptide sensor
Source: RSC Adv. 2026 Jun 1;16(32):29361–7. doi: 10.1039/d6ra00876c (PMC13227483; doi:10.1039/d6ra00876c)
Supplement: RA-016-D6RA00876C-s001 [file RA-016-D6RA00876C-s001.pdf]

Supplementary Information (SI) for RSC Advances.
This journal is © The Royal Society of Chemistry 2026

ELECTRONIC SUPPORTING INFORMATION

Selective and Sensitive Recognition of Zn^{2+} by a Dansyl-Derived Peptide Sensor

Alexandre Bianchi [a], Miriam Gaal [a], Priscilla S. Brunetto [a], Claudia Tringali [a], and Katharina M. Fromm [a]*

[a] Univ. Fribourg, Department of Chemistry and National Center of Competence in Research Bio-inspired Materials, Chemin du Musée 9, CH-1700 Fribourg, Switzerland.

E-mail: katharina.fromm@unifr.ch

Table of contents

HPLC: Retention times of D_1 3

Electrospray ionization mass spectrometry of D_1 4

Electrospray ionization mass spectrometry of D_1-Zn^{2+} complex 4

Various study of D_1 at λ_{ex} = 340 nm 5

Study of selectivity 5

Study of various Zn^{2+} counterion salts 5

Study of interference by various metal ions 6

pH study 6

Study of the binding interactions between D_1 and Zn^{2+} 7

Determination of the binding constant between D_1 and Zn^{2+} 7

Lifetime measurements of D_1 and D_1-Zn^{2+} complex 8

Limit of detection 8

Fluorescence titrations 9

HPLC: Retention times of D_1

Table S1 Retention time [min] of D_1 using semi-preparative reverse phase HPLC with a linear gradient from 95% to 70% of A in B, at a flow rate of 5 mL/min for 25 min, where A is a solution of 0.1% TFA in H_2O, and B is a solution of 0.1% TFA in ACN.

**Model**	**Retention time [min]**
Dansyl-HGHW-NH_2 (D_1)	17.90

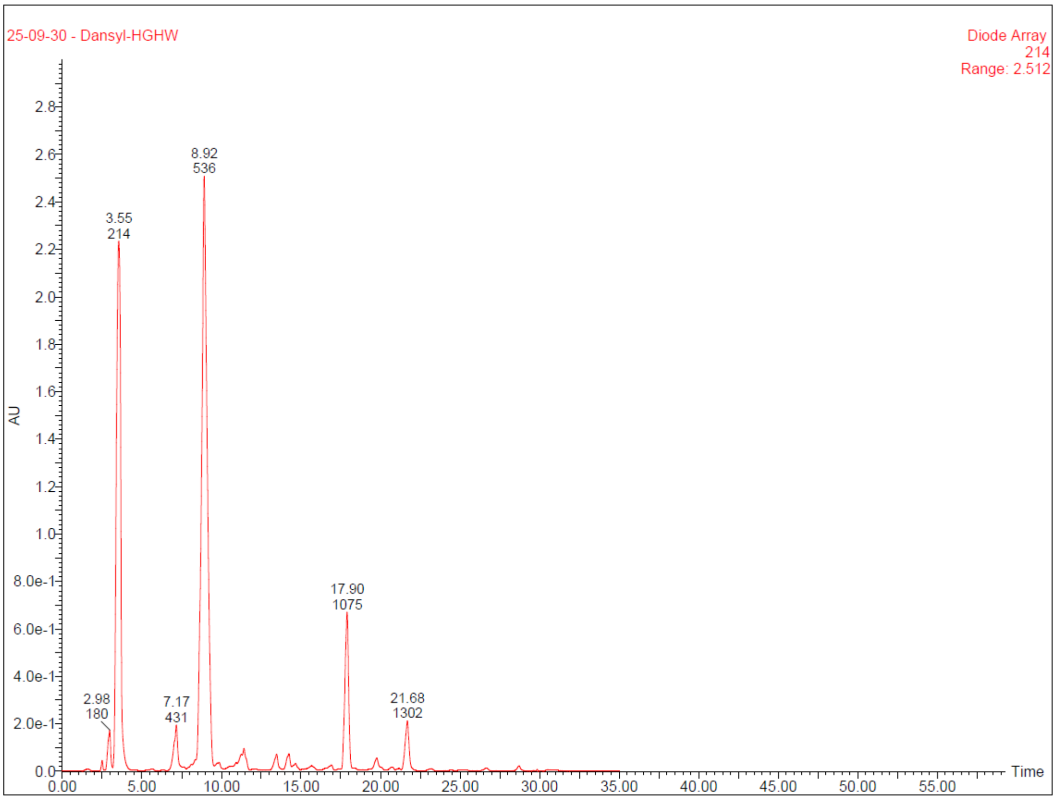

Fig. S1 HPLC chromatogram of Dansyl-HGHW (D_1).

Electrospray ionization mass spectrometry of D_1

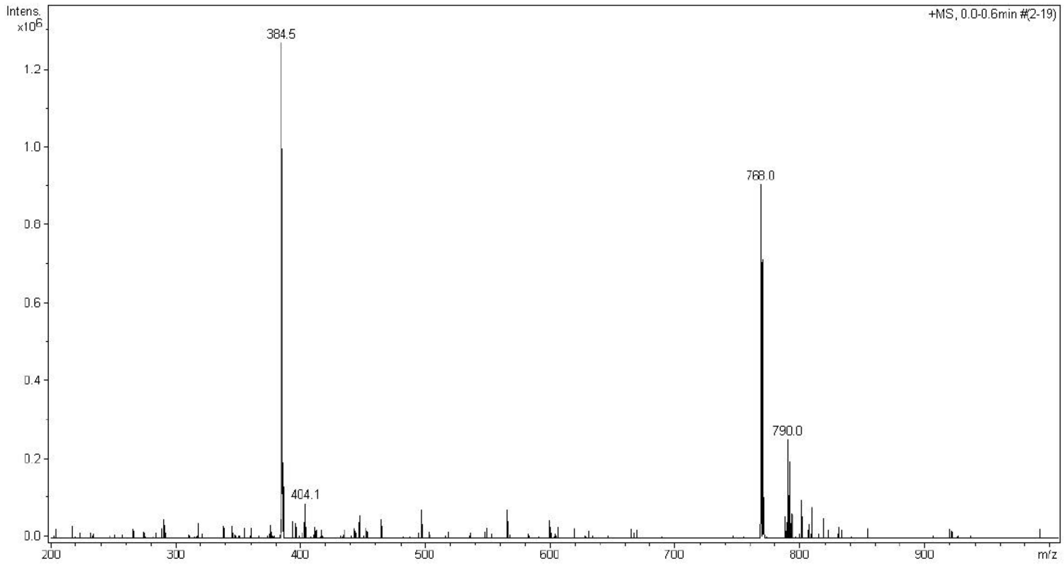

Fig. S2 ESI-MS spectrum of Dansyl-HGHW (D_1). $[M+H]^+_{calc}$ (m/z) : 767.3 ; $[M+H]^+_{exp}$ (m/z) : 768.0 ; $[M+Na]^+_{exp}$ (m/z) : 790.0 ; $[M+2H]^{2+}_{exp}$ (m/z) : 384.5.

Electrospray ionization mass spectrometry of D_1-Zn^{2+} complex

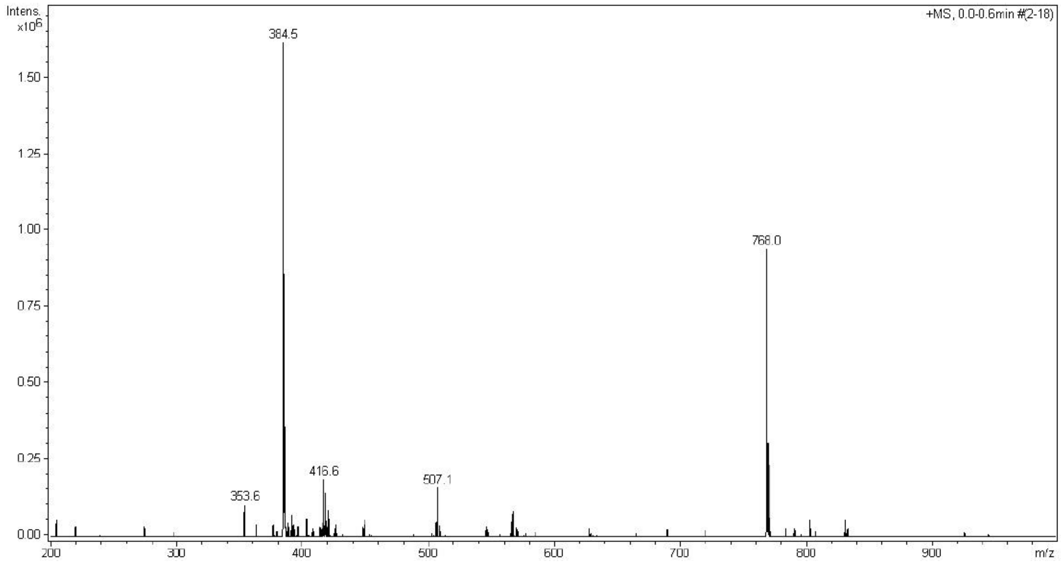

Fig. S3 ESI-MS spectrum of Dansyl-HGHW (D_1) in the presence of an excess of Zn^{2+}. $[M+H]^+_{calc}$ (m/z) : 767.3 ; $[M+H]^+_{exp}$ (m/z) : 768.0 ; $[M+Zn]^{2+}_{exp}$ (m/z) : 416.6 ; $[M+2H]^{2+}_{exp}$ (m/z) : 384.5.

Various study of D_1 at λ_{ex} = 340 nm

Study of selectivity

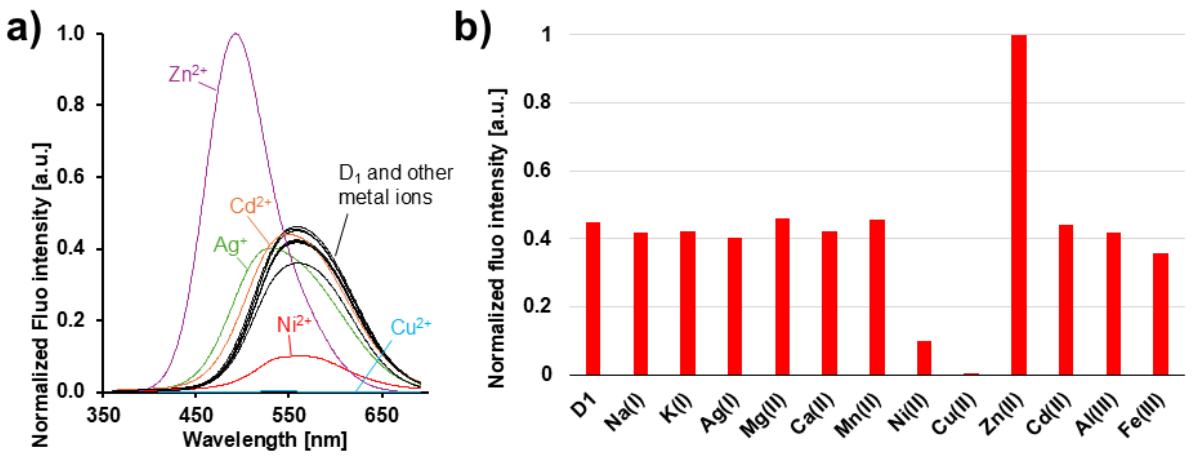

Fig. S4 a) Voigt-deconvoluted and normalized fluorescence spectra of D_1 (1×10^{-5} M) in HEPES buffer (20 mM, pH 7.4-7.5) at 25°C, λ_{ex} = 340 nm. D_1 alone (green bar), D_1 + 3 eq. of the indicated metal ions (red bars). b) Corresponding histogram.

Study of various Zn^{2+} counterion salts

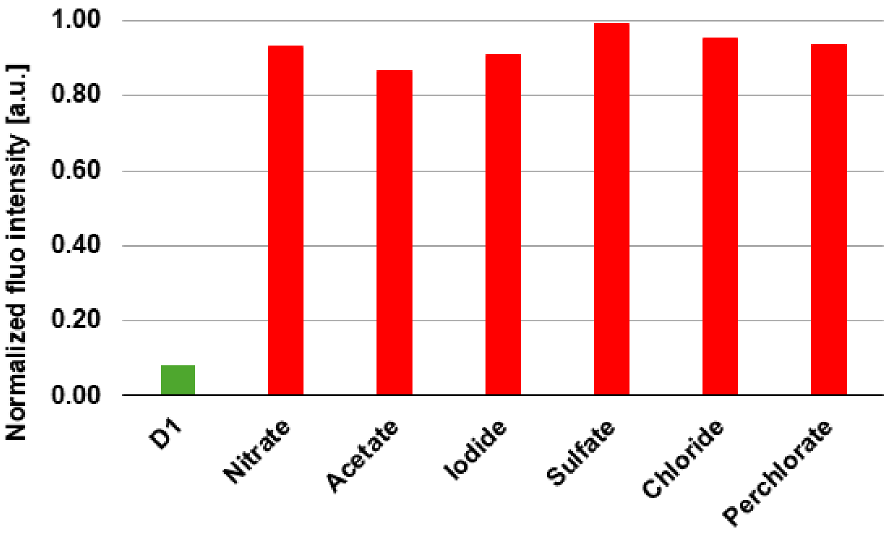

Fig. S5 Study of various Zn^{2+} counterion salts on D_1 (1×10^{-5} M) in HEPES buffer (20 mM, pH 7.4-7.5) at 25°C, λ_{ex} = 340 nm. D_1 alone (green bar), and D_1 + 3 eq. of the indicated Zn^{2+} salts (red bars).

Study of interference by various metal ions

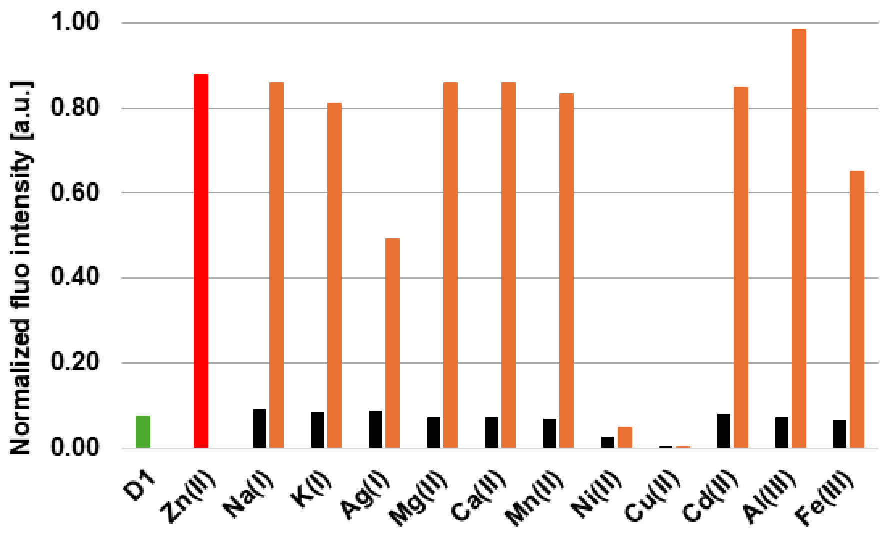

Fig. S6 Interference study of various metal ions on D_1 (1×10^{-5} M) in HEPES buffer (20 mM, pH 7.4-7.5) at 25°C, λ_{ex} = 340 nm. D_1 alone (green bar), D_1 + 3 eq. of Zn^{2+} (red bar), D_1 + 3 eq. of the indicated metal ions (black bars), then addition of 3 eq. of Zn^{2+} (orange bars).

pH study

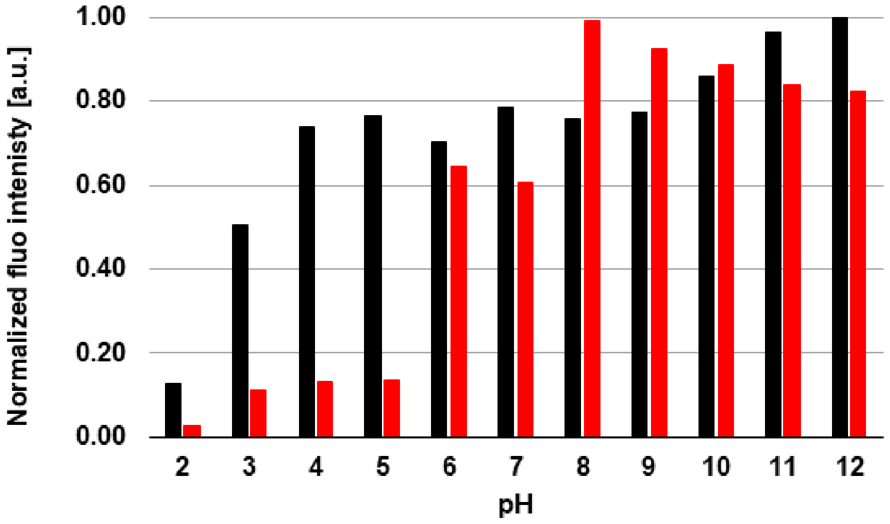

Fig. S7 pH-dependent fluorescence response of D_1 (1×10^{-5} M) at 25°C, λ_{ex} = 340 nm. Black bars represent D_1 alone. Red bars represent D_1 + 3 eq. of Zn^{2+}. The pH was adjusted using HNO_3 and NaOH solutions, both at 0.1 M.

Study of the binding interactions between D_1 and Zn^{2+}

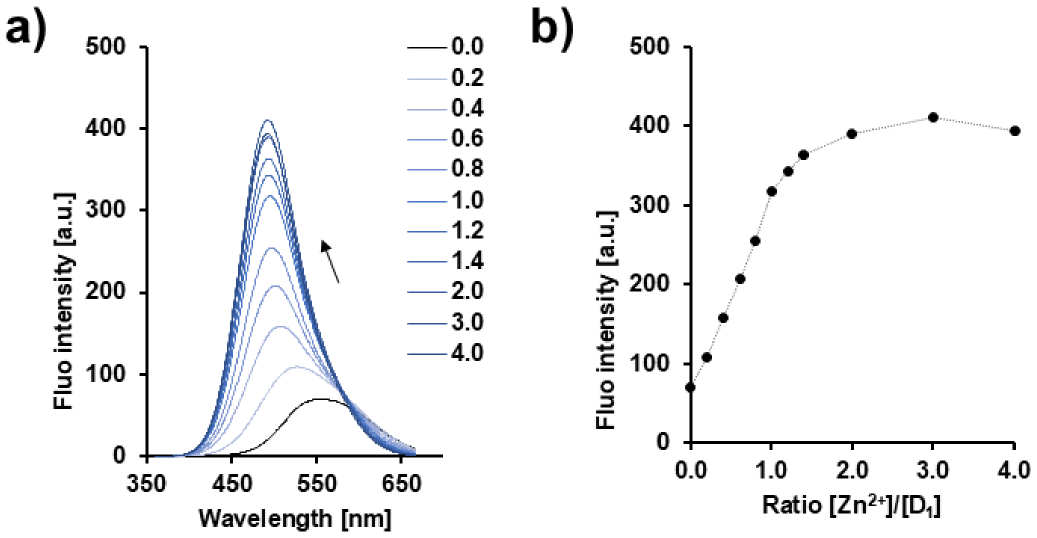

Fig. S8 a) Voigt-deconvoluted fluorescence titration spectra D_1 (1×10^{-5} M) in HEPES buffer (20 mM, pH 7.4-7.5) at 25°C, λ_{ex} = 340 nm, recorded with increasing amount of Zn^{2+} (from 0 to 4.0 eq.). b) Plot of fluo intensity as a function of Zn^{2+} equivalents.

Determination of the binding constant between D_1 and Zn^{2+}

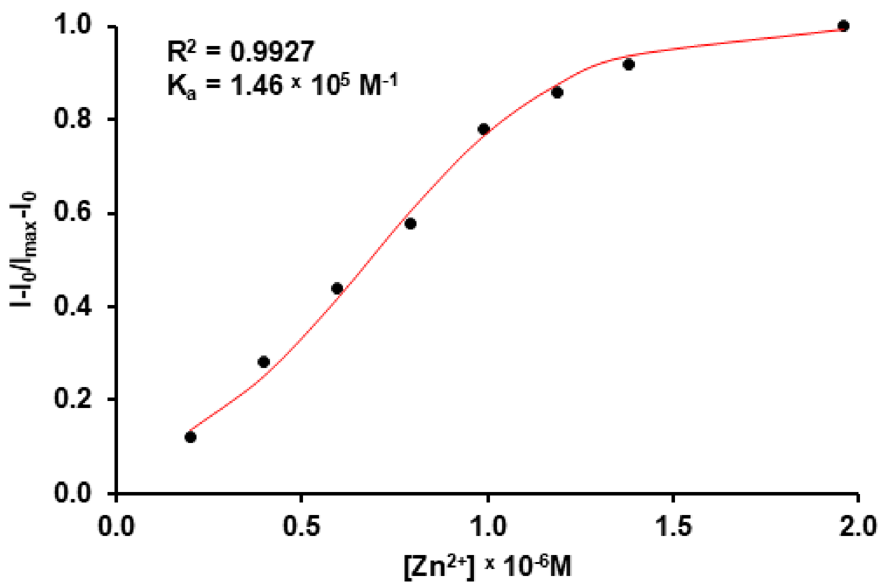

Fig. S9 Fitting curve of $I\text{-}I_0/I_{max}\text{-}I_0$ versus the amount of Zn^{2+} to determine the binding constant of the 1:1 complex.

Lifetime measurements of D_1 and D_1-Zn^{2+} complex

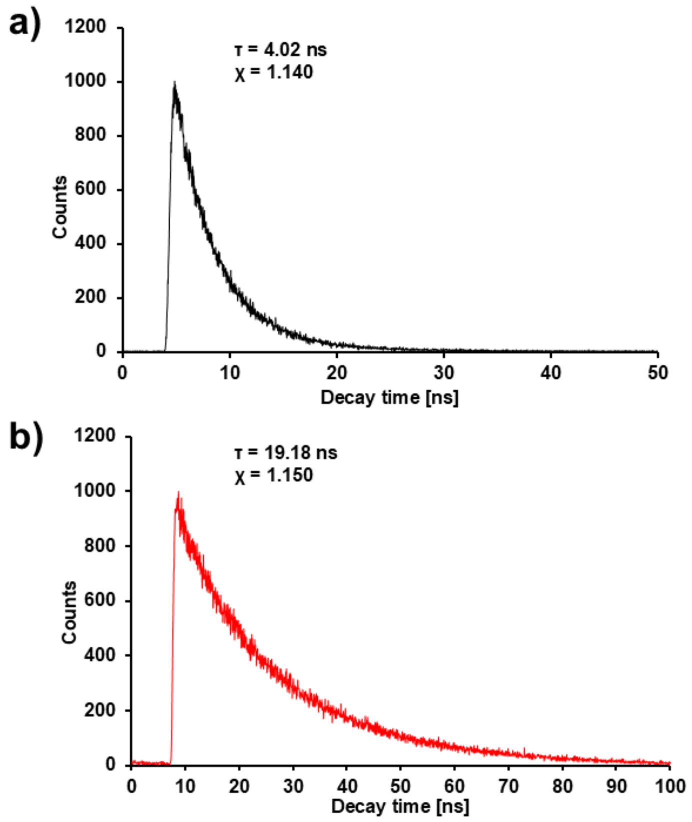

Fig. S10 Fluorescence lifetime measurements at 25°C, λ_{ex} = 405 nm. a) D_1 b) D_1-Zn^{2+} complex.

Limit of detection

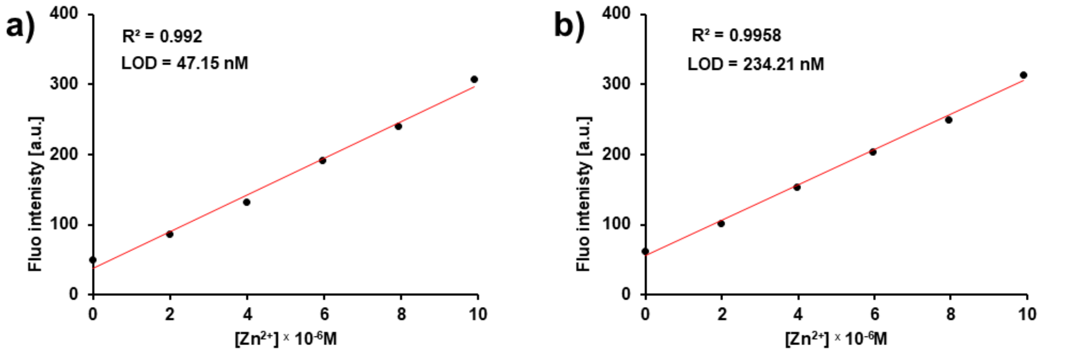

Fig. S11 Standard deviation and linear fitting to determine the limit of detection of D_1 a) λ_{ex} = 290 nm b) λ_{ex} = 340 nm.

Fluorescence titrations

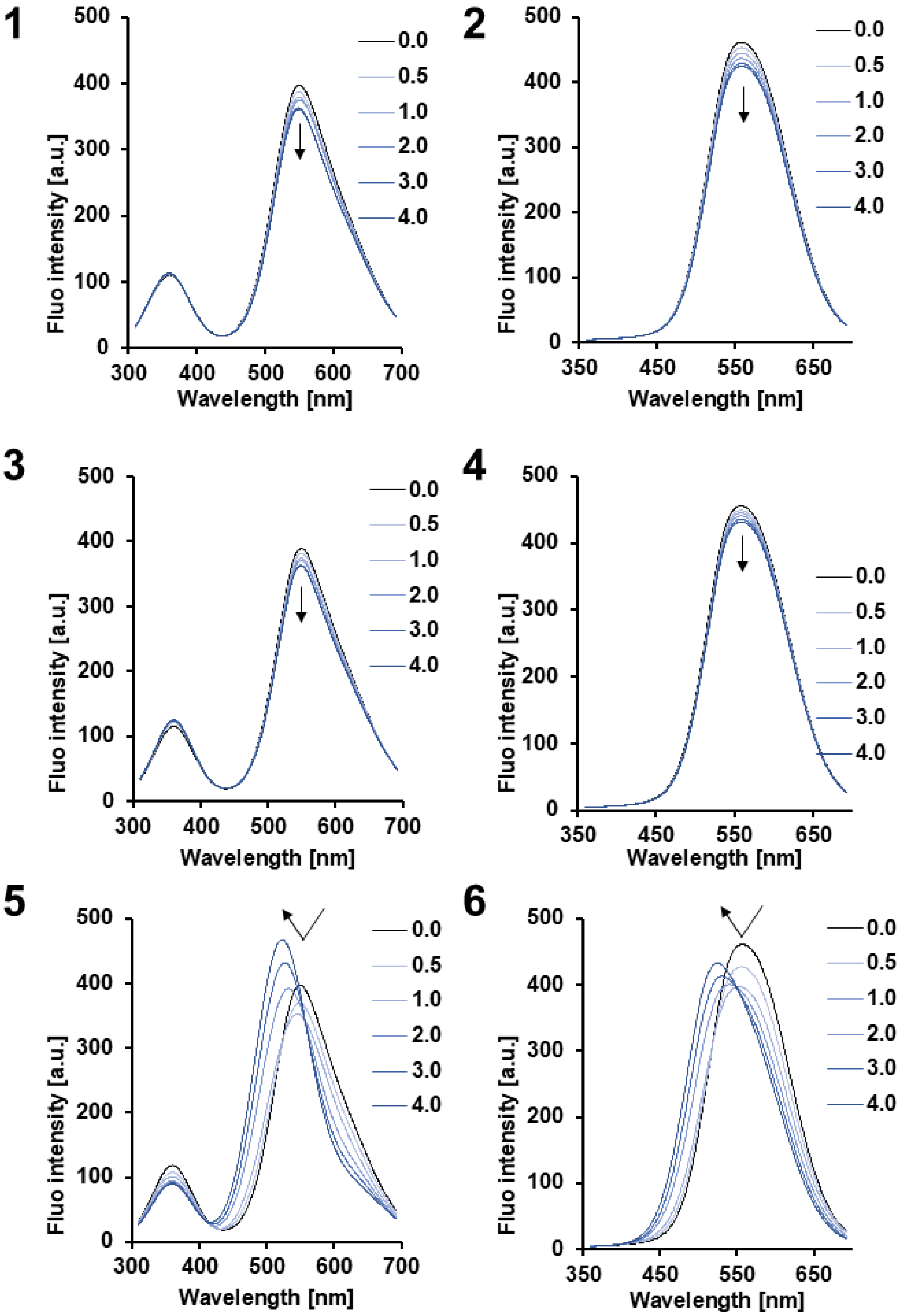

Fig. S12 Voigt-deconvoluted fluorescence spectra of D_1 (1×10^{-5} M) in HEPES buffer (20 mM, pH 7.4-7.5) at 25°C upon addition of various nitrate salt solutions. (Odd-numbered spectra) λ_{ex} = 290 nm. (Even-numbered spectra) λ_{ex} = 340 nm. Spectra 1 & 2: Na^+; 3 & 4: K^+; 5 & 6: Ag^+.

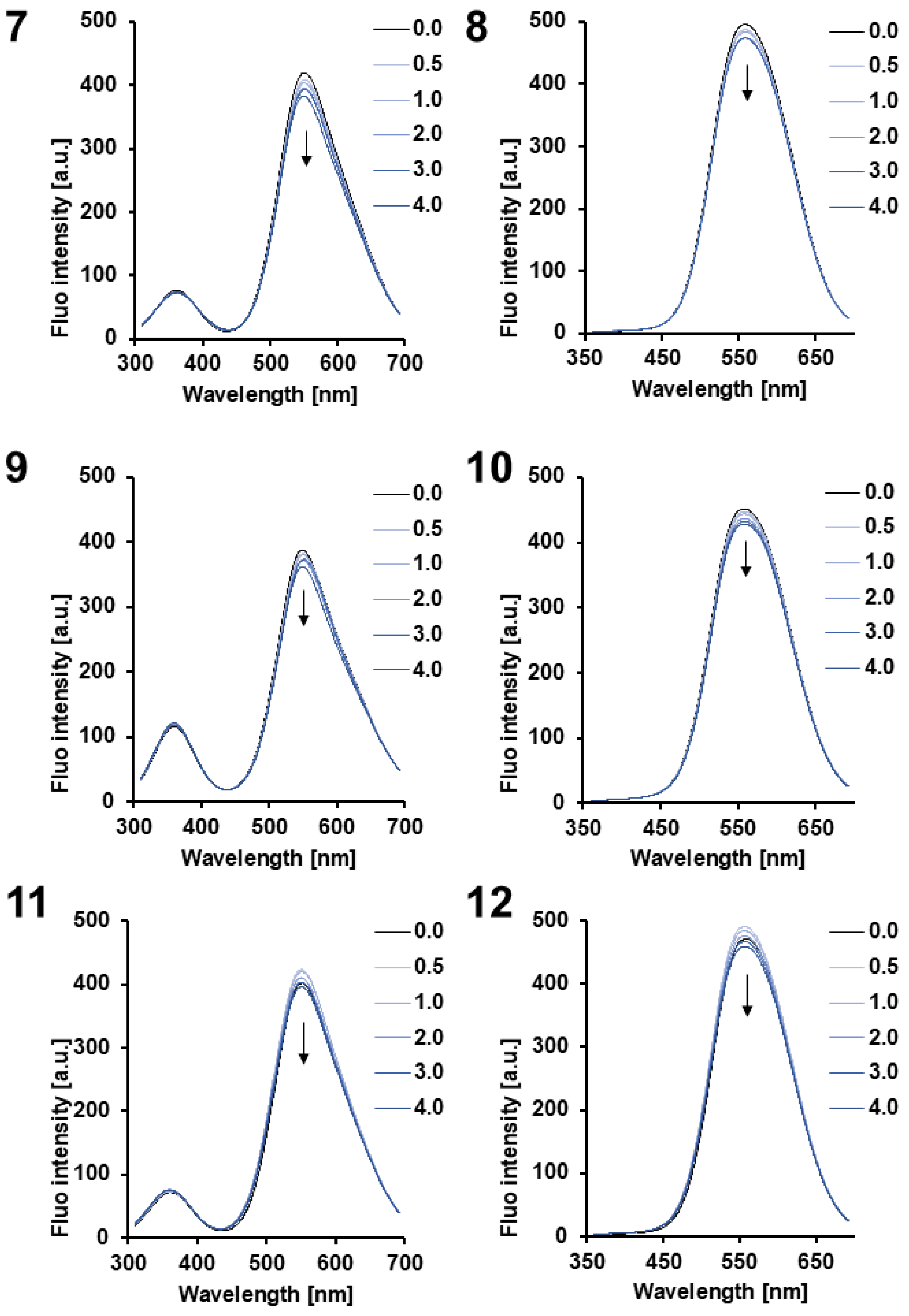

Fig. S13 Voigt-deconvoluted fluorescence spectra of D_1 (1×10^{-5} M) in HEPES buffer (20 mM, pH 7.4-7.5) at 25°C upon addition of various nitrate salt solutions. (Odd-numbered spectra) λ_{ex} = 290 nm. (Even-numbered spectra) λ_{ex} = 340 nm. Spectra 7 & 8: Mg^{2+}; 9 & 10: Ca^{2+}; 11 & 12: Mn^{2+}.

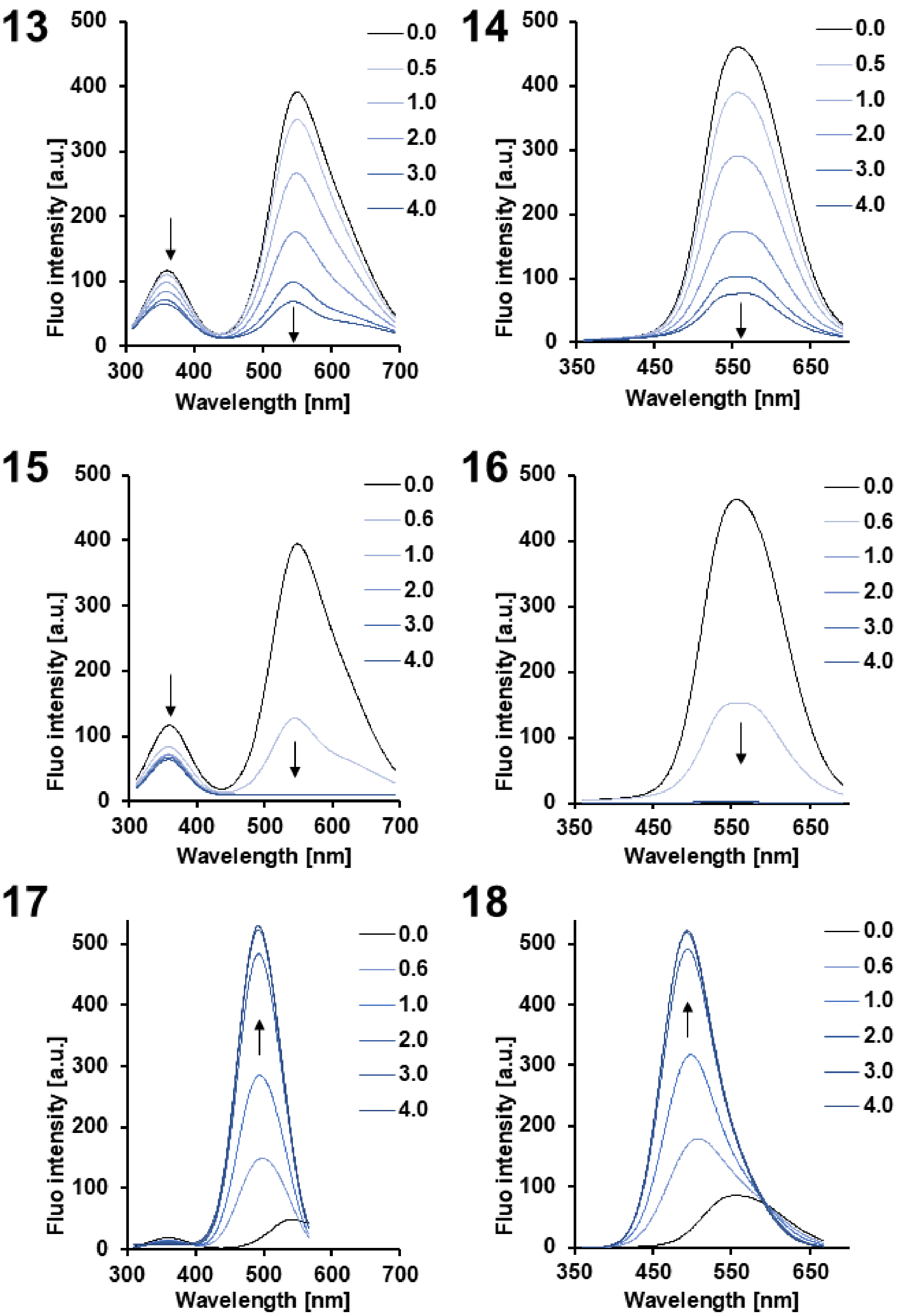

Fig. S14 Voigt-deconvoluted fluorescence spectra of D_1 (1×10^{-5} M) in HEPES buffer (20 mM, pH 7.4-7.5) at 25°C upon addition of various nitrate salt solutions. (Odd-numbered spectra) λ_{ex} = 290 nm. (Even-numbered spectra) λ_{ex} = 340 nm. Spectra 13 & 14: Ni^{2+}; 15 & 16: Cu^{2+}; 17 & 18: Zn^{2+}.

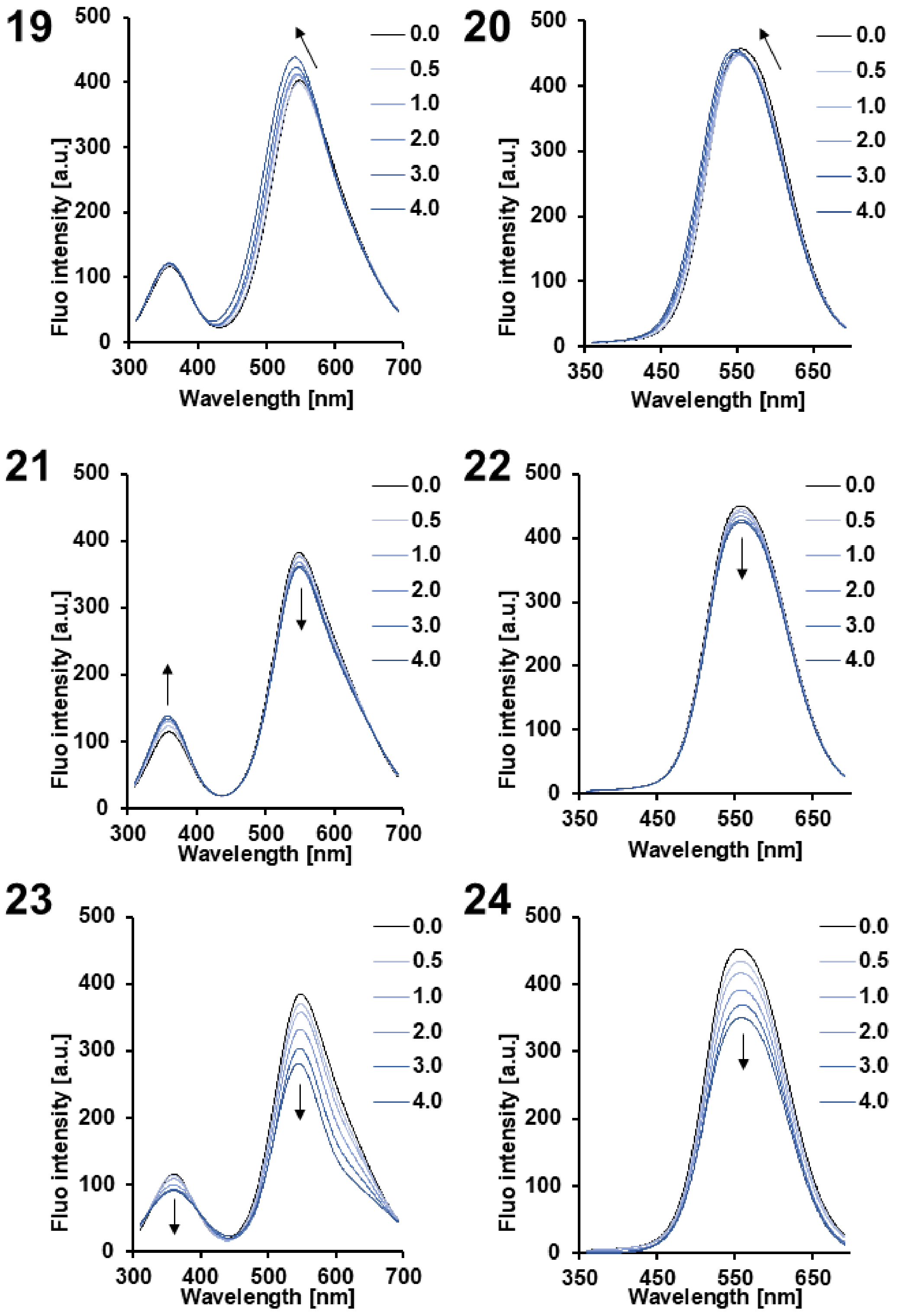

Fig. S15 Voigt-deconvoluted fluorescence spectra of D_1 (1×10^{-5} M) in HEPES buffer (20 mM, pH 7.4-7.5) at 25°C upon addition of various nitrate salt solutions. (Odd-numbered spectra) λ_{ex} = 290 nm. (Even-numbered spectra) λ_{ex} = 340 nm. Spectra 19 & 20: Cd^{2+}; 21 & 22: Al^{3+}; 23 & 24: Fe^{3+}.
